# Allelic Variation at the *Rht8* Locus in a 19th Century Wheat Collection

**DOI:** 10.1100/2012/385610

**Published:** 2012-04-30

**Authors:** Linnéa Asplund, Matti W. Leino, Jenny Hagenblad

**Affiliations:** ^1^Department of Ecology and Evolution, Uppsala University, SE-752 36 Uppsala, Sweden; ^2^Department of Crop Production Ecology, Swedish University of Agricultural Sciences, SE-750 07 Uppsala, Sweden; ^3^Swedish Museum of Cultural History, SE-643 98 Julita, Sweden; ^4^IFM Molecular Genetics, Linköping University, SE-581 83 Linköping, Sweden; ^5^Department of Biology, Norwegian University of Science and Technology, NO-7491 Trondheim, Norway

## Abstract

Wheat breeding during the 20th century has put large efforts into reducing straw length and increasing harvest index. In the 1920s an allele of *Rht8* with dwarfing effects, found in the Japanese cultivar “Akakomugi,” was bred into European cultivars and subsequently spread over the world. *Rht8* has not been cloned, but the microsatellite marker WMS261 has been shown to be closely linked to it and is commonly used for genotyping *Rht8*. The “Akakomugi” allele is strongly associated with *WMS261-192bp*. Numerous screens of wheat cultivars with different geographical origin have been performed to study the spread and influence of the *WMS261-192bp* during 20th century plant breeding. However, the allelic diversity of WMS261 in wheat cultivars before modern plant breeding and introduction of the Japanese dwarfing genes is largely unknown. Here, we report a study of WMS261 allelic diversity in a historical wheat collection from 1865 representing worldwide major wheats at the time. The majority carried the previously reported 164 bp or 174 bp allele, but with little geographical correlation. In a few lines, a rare 182 bp fragment was found. Although straw length was recognized as an important character already in the 19th century, *Rht8* probably played a minor role for height variation. The use of WMS261 and other functional markers for analyses of historical specimens and characterization of historic crop traits is discussed.

## 1. Introduction

During the green revolution in 1960's and 1970's the yield of cereal grain increased dramatically and annual production doubled [1]. This was partly due to changed cultivation practices but primarily a result of the development of new varieties of wheat, corn, and rice. One important aspect of the new varieties was the shorter, sturdier straw that could take large amounts of fertilizers without suffering from lodging.

The reduction in straw length was a result of cultivars being insensitive to gibberellin [2]. For example, Peng et al. [3] reported that the mutant alleles of the genes *reduced height-1, (Rht-B1 and Rht-D1) *leading to dwarfism in wheat, as well as the maize gene *dwarf-8 *(*d8*), are orthologues of the *Arabidopsis Gibberellin Insensitive (GAI) *gene. Unfortunately the two *Rht* genes, *Rht-B1 *and *Rht-D1, *also reduce seedling establishment and coleoptile length under some environmental conditions.

Such negative effects on seedling vigour have not been found in another semidwarfism gene, *Rht8* [13], located on chromosome 2D [14]. Although the molecular identity of *Rht8 *is still unknown, Korzun et al. [15] showed a close association with the microsatellite marker WMS261. Several alleles of this marker exist and the 164 bp allele increased height with 3 cm compared to the 174 bp allele, while the 192 bp allele, diagnostic for the semidwarf phenotype, was associated with a reduction in 7-8 cm compared to the 174 bp allele.

A few exceptions to the linkage between WMS261 and *Rht8* have been reported [16]. Nevertheless, WMS261 has been useful in a large number of screens for *Rht8* polymorphisms in various materials (Table 1). The dwarfing allele of *Rht8* and associated *WMS261-192 bp *was introduced from the Japanese variety “Akakomugi” through Italian breeding programs in the 1920's [4]. After that, it was used in several crossings and spread to the rest of the world [17]. In southern and central Europe, this allele is now very abundant [4, 6] and it is found almost exclusively in certain areas like Ukraine [5] and Bulgaria [11]. Additionally, in China the *WMS261-192 bp* is very common [10]. Interestingly, *WMS261-192 bp* is also found in several Chinese landraces suggesting an alternative source of *Rht8* in Chinese cultivars to the “Akakomugi”-Italian breeding origin [12]. The semidwarf CIMMYT varieties usually carry the *WMS261-164 bp* allele [4]. These lines have reduced height through *Rht-B1b *and *Rht-D1b*. Worland et al. [4] speculate that addition of *Rht8* would lead to a too strong dwarfing phenotype. In varieties from USA, UK, Germany, and France *WMS261-174 bp* is the most common allele. This was suggested to be due to its linkage with the photoperiod sensitive *Ppd-D1b* allele that might be beneficial for northern varieties [4, 6].

In spite of these extensive screenings, the world-wide distribution of WMS261 alleles in the era before modern plant breeding as well as introduction of the “Akakomugi” allele is unknown. The objective of this study is to explore the presence of the different WMS261 alleles in a historic 19th century material. Although no formal plant breeding (i.e., planned crossings and pedigree-based selections) took place in the 19th century, numerous well-characterized wheat cultivars existed [18, 19]. These, more or less, pure lines derived from landraces were multiplied and sold by seed companies and were thus spread and cultivated over large areas.

Several of the most recognized wheat cultivars in the 1860s were displayed at the International Exhibition in London 1862. Seed samples from the exhibition were taken to Stockholm, Sweden where they, together with some German cultivars, were multiplied at the Experimental Field of The Royal Academy of Agriculture during subsequent years [20]. Samples of the harvest of 1865 were saved in glass containers and stored at the academy museum for 100 years before being moved to the Swedish Museum of Cultural History where the samples have been kept since. Here, we report on the WMS261 genotyping of these 147-year-old seeds and the possible influence of *Rht8* in 19th century wheats.

## 2. Material and Methods

### 2.1. Historical Plant Material

Fifty-nine historical wheat varieties, harvested in 1865, were obtained from the seed collection of The Royal Swedish Academy of Forestry and Agriculture (Table 2). The seeds in this seed collection are no longer viable but genetic analysis of the aged DNA is possible [22]. Information regarding sample origin, cultivar origin, and subspecies (Table 2) was gathered from the archives of The Royal Swedish Academy of Forestry and Agriculture and complemented with data from 19th century literature on cereal cultivation [18–21]. Data on straw length (Table 2) and lodging resistance in test cultivations 1865 was taken from Juhlin-Dannfelt [21].

### 2.2. Molecular Analysis

DNA extractions of historical material were made at Linköping University in a laboratory where cereal DNA work is not regularly performed. DNA was extracted from single seeds using the FastDNA SPIN Kit and FastPrep Instrument (MP Biomedicals), with extraction blanks performed in parallel as negative controls.

*Rht8* was genotyped through a seminested PCR for the marker WMS261. The primer pair Rht8f (TGTAAAACCACGGCCAGTCTCCCTGTACGC) and Rht8r (CTCGCGCTACTAGCCATTG) was used for a first round of PCR, followed by a second round using a fluorescently-labelled forward primer, M13f, together with Rht8r. Each PCR reaction of 20 *μ*L consisted of 0.5 U Taq DNA Polymerase (New England BioLabs), 1X New England BioLabs ThermoPol Reaction Buffer, 0.25 *μ*M of each dNTP, 0.1 *μ*M each of the primers, and 1 *μ*L and 3 *μ*L of DNA-template for the first and the second PCR, respectively, where PCR product from the first PCR was used as template for the second. PCR amplifications were run at 3 min initial denaturation at 94°C, 30 cycles of 94°C for 20 s, 55°C for 1 min 20 s, and 72°C for 30 s and a final extension step of 72°C for 10 min. In samples failing to amplify the PCR reaction was repeated twice, the second time with an annealing temperature of 51°C to allow for annealing to mutated primer sites. Fragment lengths of PCR products were analyzed using MegaBACE 1000 (Amersham Biosciences) and MegaBACE Fragment Profiler version 1.2

## 3. Results

We successfully amplified the marker WMS261 in 57 out of 59 seed samples harvested in 1865 and used it as a proxy for genotyping the linked *Rht8* locus. Among the samples yielding a PCR product we found 15 accessions carrying the *WMS261-164 bp* genotype and 36 accessions with the *WMS261-174 bp* allele. Two accessions had an allele of length 182 bp. In addition four accessions were heterozygous, three for the 164, 174 genotype, and one for the 164, 182 genotype (Table 2).

For most accessions the country of origin was known. We were unable to detect any clear pattern with respect to country of origin and *Rht8* genotype. In most countries from which we had more than one accession both the *WMS261-164 bp* and the *WMS261-174 bp* allele were present. The exceptions were Hungary (all three *WMS261-174 bp*), Sweden (both *WMS261-174 bp*) and the US (both *WMS261-164 bp*). All spelt wheats studied, except one, carried the *WMS261-174 bp* allele.

We evaluated data on straw length from the test cultivations performed in 1865, the cultivations from which the seeds were taken. Data was available for 41 cultivars and straw lengths ranged from 91 to 127 cm. We found no correlation between straw length and the two WMS261-genotypes, -164 bp and -174 bp (two sample *t*-test, *df* = 34, *P* = 0.78). The degree of lodging was registered in the cultivation records and we note that several of the tallest accessions suffer from lodging. Evidently, lodging was considered as a serious problem and tall strawwasan undesirable trait.

## 4. Discussion

The genetic diversity at the *WMS261* microsatellite has been an important diagnostic tool for genotyping the *Rht8* locus (Table 1). Previous studies have shown three different alleles, *WMS261-174 bp*, *WMS261-164 bp,* and *WMS261-192 bp*, to be internationally widespread. The majority of the accessions in our sample had either of the first two of these alleles. Some of our PCR products yielded fragments that were sized a few base pairs larger than *WMS261-164 bp* or *WMS261-174 bp*, but in accordance with Schmidt et al. [9] we did not consider them as distinct alleles, but a result of slippage or “stutter”.

Our choice of samples is in many ways comparable with those of previous studies [9, 10, 15] in that it comprises of a range of, at the time, widely cultivated and internationally representative wheat accessions. As expected the *WMS261-164 bp* and the *WMS261-174 bp* alleles were the most common ones (28 and 68% of the homozygotes, resp.). Our samples were harvested some 60 years before the first use of “Akakomugi” in crosses and the 1865 test cultivations did not include any Japanese or Chinese accessions. It is therefore not surprising that we do not detect the *WMS261-192 bp* allele.

It has been suggested that the *WMS261-174 bp* allele is linked to the *Ppd-D1b* allele and has been selected for in northern Europe. However, in contrast to screens of extant material [4, 6] we did not see the clear dominance of the *WMS261-174 bp* allele in cultivars from northern Europe and North America. In our material we found both the *WMS261-174 bp* and the *WMS261-164 bp* alleles in wheats from a wide range of countries and in many cases we found both alleles in wheats from the same country. Although all the accessions from the same country in a few cases shared the same allele we could not distinguish any clear geographic pattern in the distribution of the *WMS261-174 bp* and *WMS261-164 bp* alleles. The limited number of accessions restricts the possibility to recognize geographic patterns, but the geographic segregation of allele types [4, 6] might actually have arisen later during modern plant improvement, often based on a few key cultivars. In the cultivation data for the winter wheats flowering time (not shown) was slightly earlier for accessions with the *WMS261-174 bp* than those with the *WMS261-164 bp* allele (298 versus 300 days after sowing) but not significantly (two sample *t*-test, *df* = 27, *P* = 0.19) and did thus not show any clear support for linkage to *Ppd-D1b*.

In addition to the two major alleles (*WMS261-164 bp* and *WMS261-174 bp*), we found a few accessions with a 182 bp allele. Other studies have reported alleles differing from the three main alleles, but an allele in the 182 bp size range has only been reported previously in a single cultivar, “Madison” [7]. The wheats with the 182 bp allele was a Canadian wheat and a German wheat called “Ringelblumen” and it does not seem that they have a shared or limited origin that might otherwise have explained why the allele has been undetected in most previous studies. Unfortunately we lacked cultivation data for both the accessions homozygous for the 182 bp allele. Its correlation with a specific effect on plant height should be worthwhile investigating to further explore the relationship between different alleles at the WMS261 locus and differences in plant height.

In this study, we cannot find any correlation between genotype and plant height. The average effect of the *WMS261-174 bp* allele compared to the *WMS261-164 bp* allele is a reduction of 3 cm [15] and in the limited number of accessions this effect might be too small to detect. The test cultivations, carried out at the experimental fields of the Royal Swedish Agricultural Academy, were also performed in small and nonreplicated test plots, which further limit the possibility to reveal any effects of the *Rht8*. However, it is clear from the cultivation data that straw length and the amount of lodging were traits of concern to the 19th century plant breeders. Although *Rht8 *probably contributed little, if at all, to variation in straw length, the set of cultivars of 1865 displayed a large height range.

The cultivars studied here are from the time period when the seed industry first emerged in the Western world. Line selections from landraces with desirable traits were developed and multiplied to give rise to more uniform seed materials with more predictable traits, that is, cultivars. The most popular of these was named and described in the contemporary literature and received both national and international attention [23]. The major wheat cultivars of the 19th century have in some cases survived to the present in genebank collections, and several of the cultivars genotyped in this study can be obtained as extant material from genebanks. Most of the cultivars studied here are, however, long extinct. For these, samples from historical collections provide the only possibility to study the genetic composition of early wheat cultivars. Also for accessions still available in genebanks, there are advantages in using historical material instead. Concerns regarding the integrity of genebank material have been raised [24] and the geographic distribution of functional alleles have been shown to be much more distinct with historical than extant material [25, 26]. The specific nature of the historic specimens used here, that is, large containers with thousands of seeds [22], also permits repeated or complementary experiments.

Molecular identification of genes involved in domestication and plant improvement has recently accelerated [27, 28]. By screening the genetic diversity present and testing for selection the individual importance of different alleles can be explored. In the case of *Rht8* its role in 20th century wheat improvement is well known from extensive screens and documented crossings and pedigrees. Here we can add insight into the genetic diversity of the *Rht8* during the transition from traditional and modern agriculture, a time less well documented and more difficult to study. The use of historical and archaeological plant material [29] in addition to extant plant material can in this way help to reveal a clearer picture to the processes that formed crop plants of today.

## Figures and Tables

**Table 1:** Studies of WMS261 allelic diversity.

Reference	Plant material	Number of accessions
Worland et al. [4]	World-wide cultivars	118

Chebotar et al. [5]	Ukrainian cultivars and breeding lines	27
	US and European cultivars and breeding lines	20

Worland et al. [6]	World-wide cultivars	870

Ahmad and Sorrells [7]	Mainly US and NZ cultivars	71

Manifesto and Suárez [8]	Argentinian cultivars	165

Schmidt et al. [9]	Australian cultivars	24

Liu et al. [10]	Chinese cultivars and breeding lines	408
	CIMMYT, US and European cultivars and breeding lines	98

Ganeva et al. [11]	Bulgarian cultivars	89

Zhang et al. [12]	Chinese landraces, cultivars, and breeding lines	220

**Table 2:** Historical cultivars screened for WMS261 allelic diversity. Acc.nr refers to the seed collection inventory number in the Swedish Museum of Cultural History. Data on height and lodging are from Juhlin Dannfelt [21].

Acc.nr	Species^1^	Cultivar name	Country of origin	WMS261 allele/s	Height (cm)	Notes on lodging
NM1080	*T. ae. ae.*	Hartswood	England	164	122	Lodging
NM1081	*T. ae. ae.*	West Canada	Canada	174	91	Little lodging
NM1082	*T. ae. ae.*	Fife	Canada	164	102	
NM1083	*T. ae. ae.*	Cloves Highland	Holland	174		
NM1084	*T. ae. ae.*	Stevens	Australia	164	102	Early lodging
NM1085	*T. ae. ae.*	Hunters Winter	Germany	174	114	Lodging
NM1086	*T. ae. ae.*	Tappahannock	United States	164	119	Somewhat lodging
NM1087	*T. ae. ae.*	Richmond's Giant	England	164	114	Late lodging
NM1088	*T. ae. ae.*	Marigold	Germany	164, 174		
NM1090	*T. ae. ae.*	Red Lammas	England	174	117	Somewhat lodging
NM1091	*T. ae. ae.*	Chiddam	England	174	122	Somewhat lodging
NM1092	*T. ae. ae.*	Petticoat	Canada	174	122	
NM1093	*T. ae. ae.*	Hundredfold	England	164	112	Late lodging
NM1094	*T. ae. ae.*	Victoria	Venezuela	174	102	Somewhat lodging
NM1095	*T. ae. ae.*	Drewett's	Unknown	174	114	
NM1096	*T. ae. ae.*	Tuscany	Italy	174	114	
NM1097	*T. ae. ae.*	Hopetoun	Germany	174	117	Somewhat lodging
NM1098	*T. ae. ae.*	Southern Australia	Australia	174		
NM1099	*T. ae. ae.*	Long bearded	Unknown	164	112	Lodging
NM1100	*T. ae. ae.*	Red from Tschernigow	Ukraine	164	94	Lodging
NM1101	* T. ae.*	Summer wheat	Unknown	174		
NM1102	*T. ae. ae.*	Australia	Australia	164		
NM1103	*T. ae. ae.*	Mummy	England	164, 174	117	Somewhat lodging
NM1104	*T. ae. ae.*	Ringelblumen	Germany	182		
NM1106	*T. ae. ae.*	Red Essex	England	174	127	Somewhat lodging
NM1108	*T. ae. ae. *	Canadian	Canada	182		
NM1109	*T. ae. ae.*	Dayton	Unknown	164, 182	117	Late lodging
NM1110	*T. ae. ae.*	White Belgian	Belgium	174	114	
NM1111	*T. ae. ae.*	Hungarian	Hungary	174		
NM1112	*T. ae. sp.*	White Schwanen	Unknown	164		
NM1113	*T. ae. co.*	Igel	Switzerland	174	114	Somewhat lodging
NM1115	*T. ae. ae.*	Galizian	Poland	174	102	Lodging
NM1116	*T. ae. ae.*	Eley's Giant	Switzerland	164	99	
NM1118	*T. ae. ae.*	Sixrow	Unknown		112	Somewhat lodging
NM1120	*T. ae. ae.*	Stålvete	Sweden	174		
NM1122	*T. ae. ae.*	Nottingham	England	174	119	Much lodging
NM1123	*T. ae. ae.*	Hungarian	Hungary	174		
NM1125	*T. ae. ae.*	Three-row Chevalier	Unknown	174		
NM1126	* T. ae. ae.*	Hungarian	Hungary	174		
NM1129	*T. ae. ae.*	Sandomirka from Volhynia	Ukraine	174	117	Somewhat lodging
NM1135	*T. ae. ae.*	White Essex	England	164	112	
NM1136	*T. ae. ae.*	Probsteier	Germany	174	122	Somewhat lodging
NM1139	*T. ae. ae.*	Grano tenero	Italy	164, 174	114	lodging
NM1140	*T. ae. ae.*	Lammas	England	164	114	Lodging
NM1141	*T. ae. ae.*	Fenton	Scotland	174	102	
NM1178	*T. ae. ae.*	Bluestem	Canada	174	109	
NM1179	*T. ae. ae.*	Talavera	Spain	174	107	
NM1180	*T. ae. ae.*	Red-chaffed-pearl	United States	164	127	Lodging
NM1181	*T. ae. ae.*	Southern Australia	Australia	164		
NM1182	*T. ae. ae.*	Italian	Italy	174		
NM1186	*T. ae. ae.*	Swedish (Sammets)	Sweden	174		
NM1187	*T. ae. ae.*	Hopetoun	England	174	119	Somewhat lodging
NM1189	*T. ae. ae.*	Hickling's prolific	England	174	122	Lodging
NM1800	*T. ae. sp.*	White winter spelt	Germany	174	109	Lodging
NM1802	*T. ae. sp. *	Winter spelt	Germany/France	174		
NM1803	*T. ae. sp.*	White-club-shaped spelt	Germany/Switzerland	174		
NM1805	*T. ae. sp.*	Red winter	Germany	174	107	
NM1807	*T. ae. sp.*	Schlegel's winter	Germany		97	
NM1811	*T. ae. sp.*	White winter emma spelt	Unknown	174	107	

^1^T: Triticum, ae: aestivum, co: compactum, sp: spelta.

## References

[1] Khush GS (2001). Green revolution: the way forward. *Nature Reviews Genetics*.

[2] Hedden P (2003). The genes of the green revolution. *Trends in Genetics*.

[3] Peng J, Richards DE, Hartley NM (1999). “Green revolution” genes encode mutant gibberellin response modulators. *Nature*.

[4] Worland AJ, Korzun V, Röder MS, Ganal MW, Law CN (1998). Genetic analysis of the dwarfing gene *Rht8* in wheat—part II. The distribution and adaptive significance of allelic variants at the *Rht8* locus of wheat as revealed by microsatellite screening. *Theoretical and Applied Genetics*.

[5] Chebotar SV, Korzun VN, Sivolap YM (2001). Allele distribution at locus *WMS261* marking the dwarfing gene *Rht8* in common wheat cultivars of Southern Ukraine. *Russian Journal of Genetics*.

[6] Worland AJ, Sayers EJ, Korzun V (2001). Allelic variation at the dwarfing gene *Rht8* locus and its significance in international breeding programmes. *Euphytica*.

[7] Ahmad M, Sorrells ME (2002). Distribution of microsatellite alleles linked to *Rht8* dwarfing gene in wheat. *Euphytica*.

[8] Manifesto  MM, Suárez EY (2002). Microsatellite screening of the *Rht8* dwarfing gene in Argentinian wheat cultivars. *Annual Wheat Newsletter*.

[9] Schmidt AL, Gale KR, Ellis MH, Giffard PM (2004). Sequence variation at a microsatellite locus (XGWM261) in hexaploid wheat (*Triticum aestivum*) varieties. *Euphytica*.

[10] Liu Y, Liu D, Zhang H (2005). Allelic variation, sequence determination and microsatellite screening at the XGWM261 locus in Chinese hexaploid wheat (*Triticum aestivum*) varieties. *Euphytica*.

[11] Ganeva G, Korzun V, Landjeva S, Tsenov N, Atanasova M (2005). Identification, distribution and effects on agronomic traits of the semi-dwarfing *Rht* alleles in Bulgarian common wheat cultivars. *Euphytica*.

[12] Zhang X, Yang S, Zhou Y, He Z, Xia X (2006). Distribution of the Rht-B1b, Rht-D1b and *Rht8* reduced height genes in autumn-sown Chinese wheats detected by molecular markers. *Euphytica*.

[13] Ellis MH, Rebetzke GJ, Chandler P, Bonnet D, Spielmeyer W, Richards RA (2004). The effect of different height reducing genes on the early growth of wheat. *Functional Plant Biology*.

[14] Worland J, Law CN, Petrovic S (1990). Height reducing genes and their importance to Yugoslavian winter wheat varieties. *Savremena Poljo-Privreda*.

[15] Korzun V, Röder MS, Ganal MW, Worland AJ, Law CN (1998). Genetic analysis of the dwarfing gene (*Rht8*) in wheat. Part I. Molecular mapping of *Rht8* on the short arm of chromosome 2D of bread wheat (*Triticum aestivum* L.). *Theoretical and Applied Genetics*.

[16] Ellis MH, Bonnett DG, Rebetzke GJ (2007). A 192bp allele at the Xgwm261 locus is not always associated with the *Rht8* dwarfing gene in wheat (*Triticum aestivum* L.). *Euphytica*.

[17] Borojevic K, Borojevic K (2005). The transfer and history of “reduced height genes” (Rht) in wheat from Japan to Europe. *Journal of Heredity*.

[18] Alefeld F (1866). *Landwirtschaftliche Flora*.

[19] Körnicke F, Werner H (1885). *Handbuch des Getreidebaues*.

[20] Juhlin-Dannfelt C, Müller A

[21] Juhlin-Dannfelt C

[22] Leino MW, Hagenblad J, Edqvist J, Strese EMK (2009). DNA preservation and utility of a historic seed collection. *Seed Science Research*.

[23] Bonjean AP, Angus WJ (2001). *The World Wheat Book: A History of Wheat Breeding*.

[24] Hagenblad J, Zie J, Leino MW Exploring the population genetics of genebank and historical landrace accessions.

[25] Jones H, Lister DL, Bower MA, Leigh FJ, Smith LM, Jones MK (2008). Approaches and constraints of using existing landrace and extant plant material to understand agricultural spread in prehistory. *Plant Genetic Resources*.

[26] Lister DL, Thaw S, Bower MA (2009). Latitudinal variation in a photoperiod response gene in European barley: insight into the dynamics of agricultural spread from ’historic’ specimens. *Journal of Archaeological Science*.

[27] Doebley JF, Gaut BS, Smith BD (2006). The molecular genetics of crop domestication. *Cell*.

[28] Burger JC, Chapman MA, Burke JM (2008). Molecular insights into the evolution of crop plants. *American Journal of Botany*.

[29] Palmer SA, Smith O, Allaby RG (2012). The blossoming of plant archaeogenetics. *Annals of Anatomy*.

